# Steroidal Alkaloids from the Roots of *Veratrum mengtzeanum* Loes. with Their Anti-Inflammatory Activities

**DOI:** 10.3390/molecules28207116

**Published:** 2023-10-16

**Authors:** Wenjuan Yuan, Jinrong Ma, Xinlan Liu, Chengting Zi, Yongkai Xi, Xiaojing Shen, Guodong Li, Jun Sheng, Xuanjun Wang

**Affiliations:** 1 College of Science, Yunnan Agricultural University, Kunming 650201, China; yuanwj0805@126.com (W.Y.); zichengting@126.com (C.Z.); 2021062@ynau.edu.cn (Y.X.); 2013017@ynau.edu.cn (X.S.); 2 Key Laboratory of Pu′er Tea, Ministry of Education, Yunnan Agricultural University, Kunming 650201, China; 15752252744@163.com (J.M.); liuxinlan15@163.com (X.L.); 3 College of Food Science and Technology, Yunnan Agricultural University, Kunming 650201, China; 4 College of Chinese Medicine, Yunnan University of Chinese Medicine, Kunming 650500, China; gammar116@163.com

**Keywords:** *Veratrum mengtzeanum* Loes., steroidal alkaloids, mengtzeanines A, mengtzeanines B, anti-inflammatory activity

## Abstract

The phytochemical investigation of *Veratrum mengtzeanum* Loes. roots resulted in the isolation and characterization of two novel, namely Mengtzeanines A (**1**), Mengtzeanines B (**2**), and eight known steroidal alkaloids (**3**–**10**). Their structural properties were assessed though extensive spectroscopic techniques. All constituents **1**–**10** were analyzed for suppression of NO formation in LPS-induced RAW264.7 macrophages. Among them, constituent **6** (Verazine) showed inhibition against LPS-induced NO production (IC_50_ = 20.41 μM). Additionally, compound **6** could inhibit the secretion of IL1β, IL6, and TNFα, and downregulate the productions of iNOS and COX2 in LPS-induced RAW264.7 macrophages. Further experiments revealed that **6** exhibited a potent anti-inflammatory level in LPS-stimulated RAW264.7 macrophages via inhibiting NF-κB, and triggering of Keap1/Nrf2/HO-1 axis, implying that compound **6** may be a promising candidate for treating inflammatory disorders.

## 1. Introduction

**Figure 1:**
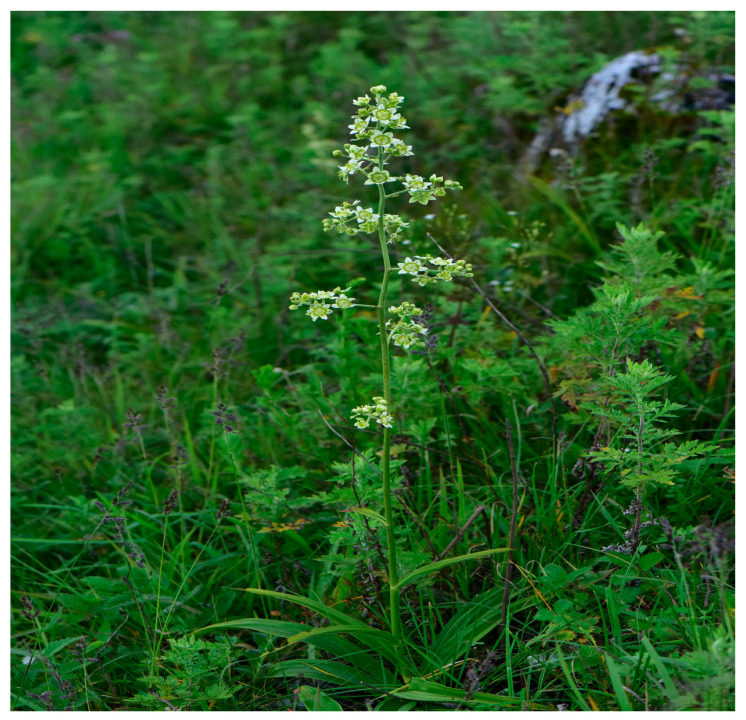
Plant material: *V. mengtzeanum* Loes.

Herbs serve as a crucial reservoir of novel drug candidates, offering a rich array of structurally unique bioactive constituents [1]. Among these, steroidal alkaloids, as secondary metabolites in plants, exhibit diverse physiological and pharmacological properties such as anti-inflammatory [1,2,3], analgesic [4,5], antibacterial [6,7], anti-tumor [8,9], and antioxidant [10,11] effects, thus representing valuable resources for drug discovery. The *Veratrum* genus comprises approximately 45 species globally, including 17 in China, and holds significance within the Liliaceae family [12,13,14]. The roots and rhizomes of *V. mengtzeanum* Loes. (Figure 1) are named “Pi Ma Cao” and are commonly employed in Traditional Chinese Medicine [15] to treat traumatic injury, swelling, and pain, which are related to inflammatory diseases [16].

Inflammation, mainly refers to a reaction of the body to defense stress caused by the stimulation of various factors, such as tissue damage and infection, which can lead to severe ailments, including cancer, diabetes, and atherosclerosis [17,18,19].

Macrophages are one of the immune cells that release reactive oxygen and nitric oxide (NO), pro-inflammatory mediators (e.g., IL6, IL1β, TNFα), damaging normal tissues and organs. Activated nuclear transcription factor κB can regulate the production of iNOS and COX-2 and suppress the release of NO, TNFα, IL1β, and IL6, offering a potential avenue for improving inflammatory diseases [19,20,21,22,23].

On the other hand, overproduction of ROS can cause oxidative stress, which leads to an inflammatory response. Heme oxygenase (HO)-1, which prevents oxidative stress, includes a protective mechanism against ROS-induced oxidative damage. The Keap1/Nrf2 axis is a crucial molecular target for inducers of antioxidant enzymes, leading to upregulation of HO-1 and decreased susceptibility to oxidative stress-associated damage, including inflammation [24,25,26,27,28].

To identify more herbs with therapeutic potential against various ailments, including those linked to inflammation and oxidative stress, we conducted a comprehensive chemical analysis of the rhizomes and roots of *V. mengtzeanum* Loes. This effort expedited the extraction of two novel Veratramine-group alkaloids, Mengtzeanines A (**1**) and B (**2**), together with eight known analogues (**3**–**10**). Furthermore, we assessed the anti-inflammatory properties of **1**–**10** through examining their suppressive consequences on LPS-induced NO formation. Additionally, we investigated the most promising compound (**6**) to unravel its anti-inflammatory mechanisms in LPS-stimulated RAW264.7 macrophages. In the current report, we present the process of extraction, structural characterization, and biological assessment of these constituents.

## 2. Results

### 2.1. Isolation and Structural Assessment

Chromatographic isolation of the acid-soluble and alkali-precipitation fractions of MeOH extract of roots and rhizomes of *V. mengtzeanum* Loes. on CC and HPLC afforded two novel alkloids—Mengtzeanines A (**1**), and Mengtzeanines B (**2**)—and eight known constituents **3**–**10** (Figure 1).

Mengtzeanines A (**1**) was obtained as a white and shapeless powder, which displayed a quasi-molecular ion [M + H]^+^ at *m*/*z* 428.3165 (calcd for C_27_H_41_NO_3_, 428.3159) in its HRESIMS spectrum, with eight magnitudes of unsaturation. The IR spectra revealed the existence of a hydroxyl group (3437 cm^−1^), and a carbonyl group (1702 cm^−1^). ^1^H-NMR spectra indicated two angular methyls [δ_H_ 0.92 (H_3_-18), and 1.19 (H_3_-19)], two secondary methyls [δ_H_ 1.43 (H_3_-21), and 0.93 (H_3_-27)], and an olefinic proton signal (OPS) at δ_H_ 5.73 (H-6). Within ^13^C-NMR spectra, 27 carbon resonances were obtained and categorized as two carbonyls at δ_C_ 214.1 (C-3), 199.6 (C-7), and olefinic bonds at δ_C_ 171.3 (C-5), and 123.8 (C-6), three quaternary carbon resonances at δ_C_ 38.6 (C-10), 43.8 (C-13), and 80.0 (C-20), and four methyl resonances at δ_C_ 13.4 (C-18), 17.3 (C-19), 24.7 (C-21), and 16.5 (C-27). These spectroscopic data indicated a Veratramine-type alkaloid similar to stenophylline B [29]. A noteworthy distinction was the presence of an additional methylene resonance, suggesting the possible presence of an extra ketocarbonyl group.

**Figure 2:**
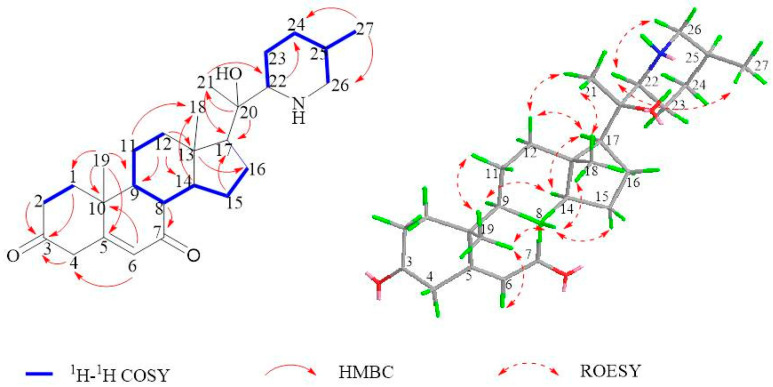
Key COSY (bold), HMBC (arrows), and ROESY (arrows) correlations of compound **1**.

Within the HMBC spectra of **1** (Figure 2), the relationships of δ_H_ 5.73 (H-6) with δ_C_ 40.3 (C-4), 171.3 (C-5), 199.6 (C-7), 38.6 (C-10), and of δ_H_ 1.73(H-8), 0.94 (H-9), and 1.19 (H_3_-19) to δ_C_ 199.6 (C-7) evidenced that the carbonyl group was positioned at the C-7. The corresponding stereochemistry of **1** was analyzed through NOESY experiments (Figure 2), the NOE relations of δ_H_ 1.19 (CH_3_–19)/1.73 (H-8), 1.73 (H-8)/0.92 (CH_3_–18), 0.92 (CH_3_–18)/1.43 (CH_3_–21), and 1.43 (CH_3_–21)/0.93 (CH_3_–27), suggesting the same β-orientations of CH_3_–18, CH_3_–21, CH_3_–28, and H-8; whereas α-orientations of H-17, H-14, and H-9 were in agreement with the configurations found in stenophylline B and similar to those in Veratramine. Based on these findings, **1** was deduced as depicted and subsequently named Mengtzeanines A. 

**Figure 3:**
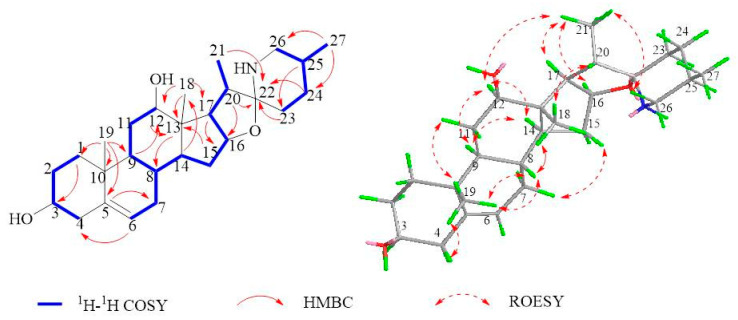
Key COSY (bold), HMBC (arrows), and ROESY (arrows) correlations of compound **2**.

Mengtzeanines B (**2**) was a white and shapeless powder, with a molecular formula of C_27_H_42_NO_3_, in accordance with the HRESIMS ion at *m*/*z* 430.3317 [M + H]^+^ (calcd for C_27_H_43_NO_3_, 430.3316), matching seven unsaturation indices. IR absorption for hydroxyl (3436 cm^−1^), and olefinic (1460 and 1379 cm^−1^) functions was observed. ^1^H, ^13^C-NMR, and HSQC spectral data of **2** revealed 27 carbon indicators (Table 1, Figure 3). ^1^H-NMR spectra demonstrated two angular methyls at δ_H_ 0.87 (H-18), and 1.01 (H-19), two secondary methyls at δ_H_ 0.98 (H-21), and 0.85 (H-27), and an OPS at δ_H_ 5.34 (H-6). Within ^13^C-NMR spectra, 27 carbon resonances were obtained and categorized as one pair of olefinic bonds at δ_C_ 140.6 (C-5), and 121.5 (C-6), three quaternary carbon resonances at δ_C_ 36.3 (C-10), 44.7 (C-13), and 99.2 (C-22), three heteroatomic methines at δ_C_ 71.6 (C-3), 72.3 (C-12), and 77.9 (C-16), and four methyl resonances at δ_C_ 17.4 (C-18), 19.3 (C-19), 15.7 (C-21), and 19.4 (C-27). These spectroscopic data were identical to those of **2**; the primary slasodine [30] distinction came from C-12 (Δδ_C_ 7.4 ppm), which strongly suggested a different absolute configuration at the 12-OH position. Furthermore, the close carbon chemical shift values of C-11, C-13, and C-18 were between **2** and implied that **2** featured a 12-βOH configuration. The ROESY relationships (Figure 3) between H-12/H-15 and H-15/H-17 in **2** further supported the β configuration at 12-OH. Drawing from these findings, the structure of **2** was designated as Mengtzeanines B. All dates positions are shown in Supplementary Figures S1.1–S2.9.

### 2.2. Structural Assessment of Known Constituents

The known constituents were determined by juxtaposing spectroscopic and physical findings (^1^H-NMR, ^13^C-NMR, DEPT, and MS) with the published reports, and this included 3-Angeloylzygadenine (**3**) [31], 3-Veratroylgemine (**4**) [32], Jervine (**5**) [33], Verazine (**6**) [34], Vermitaline (**7**) [35], 3-Angeloylzygadenine-β-N-oxide (**8**) [36], Cyclopamine (**9**) [37], Veratramine (**10**) [38].

### 2.3. Biological Studies

#### 2.3.1. Anti-Inflammatory Property through the Suppression of LPS-Induced NO Formation

**Figure 4:**
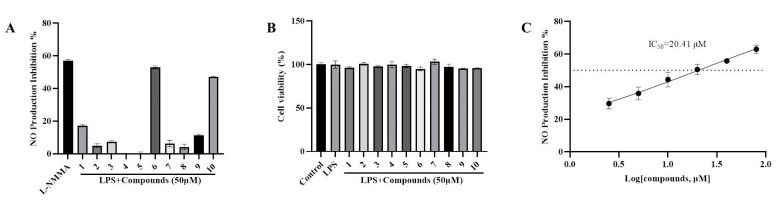
Evaluation of the anti-inflammatory properties of isolated compounds on LPS-stimulated NO generation in RAW264.7 macrophages. After pre-treatment with the tested compounds (50 μM) for 2 h, RAW264.7 macrophages were exposed to LPS for an additional 18 h. The NO content (**A**) and cell viability (**B**) were assessed with Griess reagent and MTS reagent, respectively. (**C**) The IC_50_ value of Verazine against LPS-stimulated NO generation in RAW264.7 macrophages was determined (inhibition rate reaches 50% for the dashed line). Means ± SD, *n* = 3.

To evaluate the anti-inflammatory activities of the constituents identified from the rhizomes and roots of *V. mengtzeanum* Loes., we suppressed NO generation in LPS-induced RAW264.7 cell lines. The inhibitory effects of **1**–**10** on NO production by macrophages are presented in Figure 4 and LMMA was employed as a positive control. Among the isolated constituents, **6** and **10** had a NO inhibitory effect on LPS-stimulated RAW264.7 murine macrophages (Figure 4A). At a concentration of 50 μM, compound **6** showed a similar effect to the positive control. Importantly, at a concentration of 50 μM, compound **6** did not exert any significant impact on RAW264.7 macrophage viability (Figure 4B). Compound **6**, identified as Verazine, a veratramine-type isosteroidal alkaloid category, demonstrated the most potent inhibitory activity, as evidenced by an IC_50_ value of 20.41 μM (Figure 4C), which was comparable to the positive control L-NMMA (IC_50_ = 21.86 μM). Therefore, compound **6** (Verazine) was selected for further analysis.

#### 2.3.2. Verazine Inhibits the Release of TNFα, IL1β, and IL6, and Suppresses the Production of iNOS and COX2

**Figure 5:**
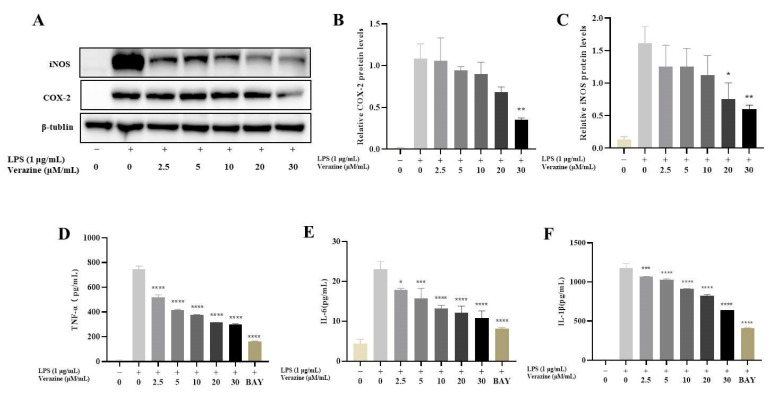
Compound **6** (Verazine) modulates the protein levels of COX2 and iNOS in LPS-exposed RAW264.7 macrophages. After pre-treatment with Verazine (2.5, 5, 10, and 20, 30 μM) for 2 h, RAW264.7 cells were exposed to LPS for an additional 18 h. The protein levels of COX2 and iNOS were detected by immunoblotting (**A**), and then quantified (**B**,**C**). The impact of Verazine on LPS-stimulated expression of TNF-α/IL-6/IL-1β in RAW264.7 macrophages. After pre-treatment with Verazine (2.5, 5, 10, 20 and 30 μM) for 2 h, RAW264.7 macrophages were stimulated with LPS for 18 h. The release of TNF-α/IL-6/IL-1β was detected through ELISA method (**D**–**F**). Means ± SD (*n* = 3). LPS; **** *p* <0.0001, *** *p* < 0.001, ** *p* < 0.01, * *p* < 0.05 against LPS alone.

To assess whether the suppressive effect of Verazine on NO formation was attributable to the downregulated translation of iNOS and COX-2, we conducted an examination of the productions of these enzymes. iNOS and COX-2 are pivotal in the pathogenesis of inflammatory diseases as they enhance vascular permeability and induce tissue damage. In our study, it was observed that Verazine significantly downregulated the productions of both iNOS and COX-2 in LPS-induced RAW264.7 macrophages (Figure 5A–C). Apart from NO, several pivotal pro-inflammatory cytokines, including TNFα, IL1β, and IL6, are involved in orchestrating inflammatory responses (including local tissue degeneration, exudation, and proliferation), causing damage to cells and tissues. The inhibition of these mediators is a promising strategy for treating inflammatory disorders. In our work, we found a dose-dependent decrease in the release of TNFα, IL1β, and IL6 in LPS-stimulated RAW264.7 macrophages following treatment with Verazine (Figure 5D–F), and BAY was employed as a positive control. These findings strongly indicate that Verazine’s anti-inflammatory effects are closely linked to its ability to suppress these pro-inflammatory cytokines.

#### 2.3.3. Verazine Suppresses the Triggering of NF-κB Signaling

**Figure 6:**
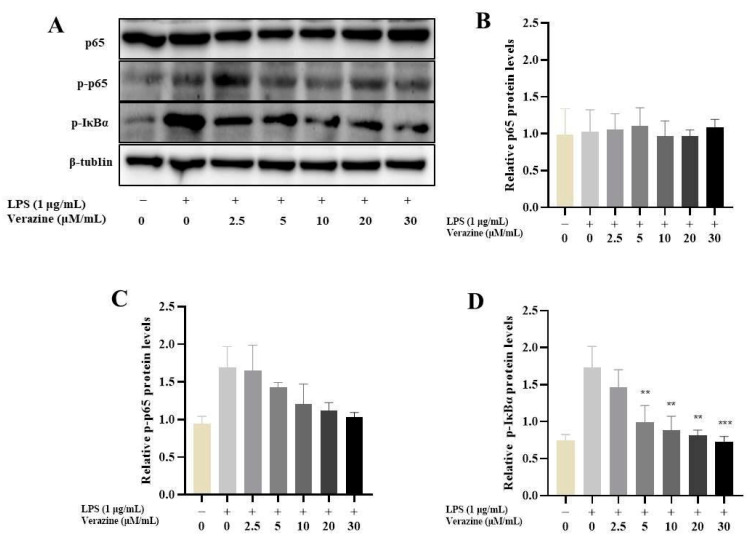
Impact of Verazine on NF-κB protein levels in LPS-exposed RAW264.7 macrophages. After pre-treatment with various concentrations of Verazine for 2 h, RAW264.7 macrophages were exposed to LPS for 16 h. The protein levels of P65 (**B**), p-P65 (**C**), and p-IκBα (**D**) were detected by immunoblotting (**A**). Mean ± SD (*n* = 3). LPS; *** *p* < 0.001, ** *p* < 0.01 against LPS alone.

The activation of NF-κB, a transcription factor comprising p65 and p50 subunits, translocates into the nucleus, and induces various pro-inflammatory mediators or cytokines, including IL6, IL1β, and TNFα, triggering a series of inflammatory responses. In order to ascertain whether Verazine’s anti-inflammatory efficacy was linked to alterations in NF-κB signaling, we conducted an initial investigation into its impact on key components, including p65, p-p65, and pIκBα (Figure 6). As anticipated, exposure to LPS led to a substantial increase in the degradation and phosphorylation of IκBα, a critical inhibitor of NF-κB activation. In contrast, treatment with Verazine (2.5–30 μM) effectively suppressed the phosphorylation and degradation of IκBα. Additionally, LPS considerably elevated the phosphorylation of NF-κB p65, a modification associated with its transcriptional activity and the initiation of NF-κB-mediated responses in macrophages. Importantly, Verazine treatment conspicuously abrogated the phosphorylation of NF-κB p65. Given NF-κB’s pivotal role in the context of inflammation, we thus postulated that Verazine’s suppression of NF-κB activity translated into the inhibition of target gene transcription, including COX-2, iNOS, IL6, IL1β, and TNFα, thereby conferring upon it a potent anti-inflammatory effect.

#### 2.3.4. Verazine Activates Nrf2/HO-1 Signaling to Alleviate Oxidative Stress in LPS-Induced RAW264.7 Macrophages

**Figure 7:**
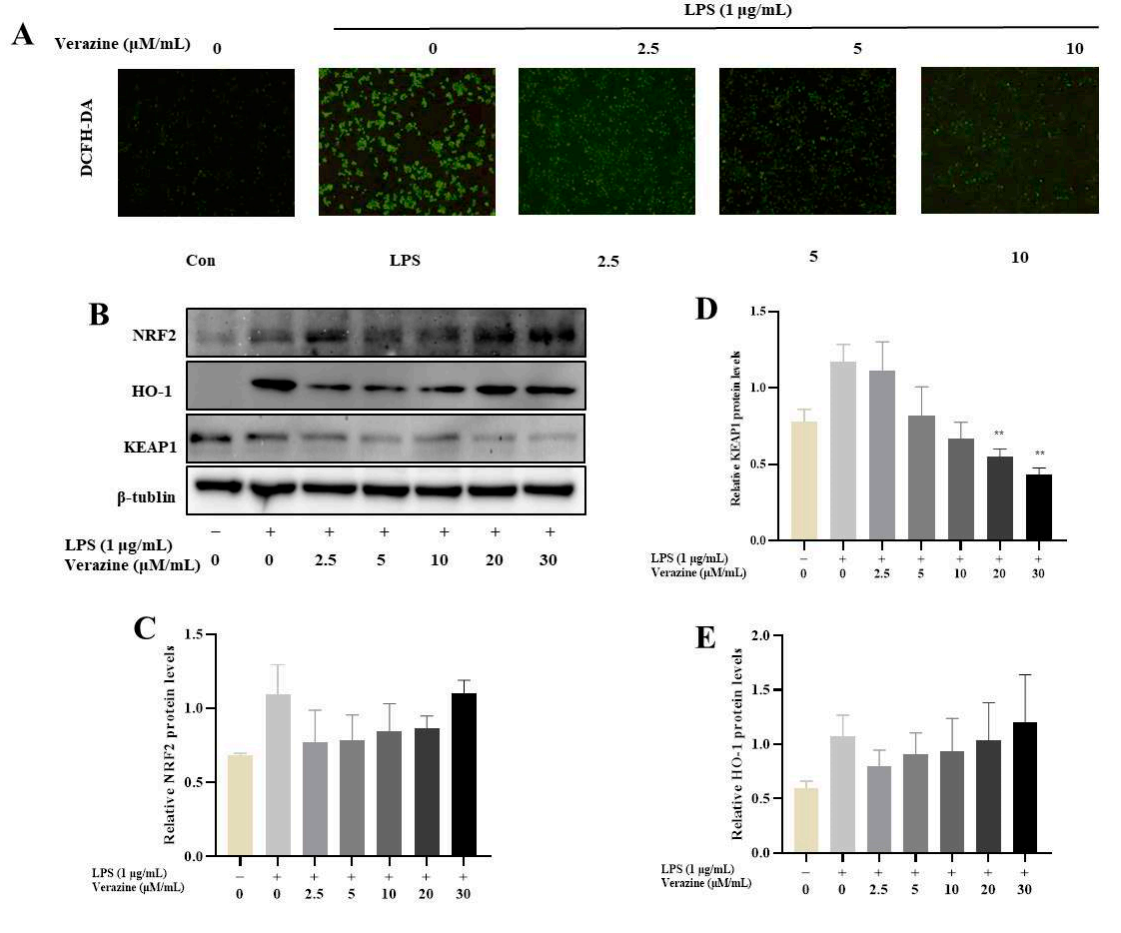
The influence of Verazine on LPS-stimulated phosphorylation/degradation of IκBα in RAW264.7 macrophages. After pre-treatment with Verazine for 2 h, RAW264.7 macrophages were exposed to LPS for 18 h. Intracellular ROS content was examined by fluorescence microscopy and flow cytometry. DCFH is marked by green fluorescence (**A**). The protein levels of Nrf2, HO-1, and Keap1 were detected by immunoblotting (**B**) and quantified (**C**–**E**). Means ± SD (*n* = 3). LPS; ** *p* < 0.01, against LPS alone.

In the aforementioned experiments, we observed that Compound **6**, also known as Verazine, exhibited a moderate inhibitory effect on the release of inflammatory factors induced by LPS. This led us to hypothesize that Verazine might play a role in mitigating LPS-mediated oxidative stress. Our observations, as depicted in Figure 7A, revealed a substantial increase in green fluorescence in RAW264.7 macrophages following LPS treatment. However, upon treatment with Verazine, the fluorescence intensity gradually diminished, indicating a significant reduction in the levels of ROS. This reduction was further confirmed through both fluorescence microscopy and flow cytometry analyses.

Nrf2 and NF-κB represent significant transcription factors responsible for governing the expression of antioxidant and pro-inflammatory genes, respectively. Oxidative stress can produce excessive ROS, leading to oxidative damage to cells, which is associated with the occurrence of inflammation disease. These findings collectively demonstrate that Verazine effectively inhibits the production and accumulation of ROS induced by LPS in RAW264.7 macrophages. Oxidative stress can be alleviated through the Nrf2/HO-1 pathway. Antioxidant molecules enter cells, Nrf2 separates from Keap1, translocates to the nucleus, and upregulates HO-1 expression, exerting an anti-inflammatory effect. In this study, Verazine can significantly reduce ROS generation in LPS-induced RAW264.7 macrophages. However, the nuclear level of Nrf2 and HO-1 was significantly increased after treatment with Verazine, while the expression of Keap1 was reduced. This suggests that Verazine alleviates oxidative stress in LPS-induced RAW264.7 macrophages via activating the Nrf2/HO-1 pathway (Figure 7B).

## 3. Materials and Methods

### 3.1. General

Polarization rotation was assessed on a Jasco P-1020 polarimeter (SHIMADZU, Kyoto City, Japan), and Shimadzu UV spectra were determined on a UV-2401A spectrophotometer (SHIMADZU, Kyoto City, Japan). The IR spectra were acquired using a Tenor 27 spectrophotometer (Bruker, Mannheim, Germany). NMR spectra were evaluated on Bruker Avance III 500/600 MHz NMR spectrometers (Bruker, Mannheim, Germany). MS-HRESI were recorded on an Agilent 1290/6540 UPLC instrument/Q-TOF-MS (Agilent, Santa Clara, CA, USA), and semi-preparative HPLC was conducted using the UV-vis detector technology of an Agilent 1200 Series chromatography system (Agilent, Santa Clara, CA, USA) equipped with a YMC-Park ODS-A (250 × 10 mm, S-5 μm) column. Column chromatography (CC) was carried out employing two distinct mesh sizes, 100–200 mesh and 300–400 mesh, RP-18 (40~63 μm, 100 Å), and Sephadex LH-20 (40~70 μm).

#### Chemicals

RAW 264.7 cells were procured from the Shanghai Cell Bank, CAS (Chinese Academy of Sciences). Dulbecco’s modified Eagle’s medium (DMEM), penicillin-streptomycin, and FBS were obtained from Hyclone (Thermo, Waltham, MA, USA). LPS (*Escherichia coli* O55:B5) and BAY (NF-kB inhibitor) were purchased from MedChemExpress (Monmouth Junction, NJ, USA). DMSO (VWR SCIENTIFIC, Santa Clara, CA, USA) Griess reagent and control drug L-NMMA were obtained from Sigma-Aldrich, Santa Clara, CA, USA. TNFα, IL1, IL1β ELISA kits and all antibodies were purchased from ABclonal, Technology (Beijing, China).

### 3.2. Plants

*Veratrum mengtzeanum* predominantly inhabits vegetation types characterized by limestone shrubland. The rhizomes and roots of *V. mengtzeanum* Loes., harvested from the Yinshan Mountains (24.492582° N, 103.166481° E), Gejiu City, Honghe Hani and Yi Autonomous Prefecture, Yunnan Province, China, in October 2020, and authenticated by Professor Guo-dong Li, YNUCM, were processed for extraction and isolation. A voucher specimen (20200012) was meticulously prepared at the Yunnan Agricultural University.

### 3.3. Extraction Process

The dried roots and rhizomes (16 kg) of *V. mengtzeanum* Loes. were crushed, and subjected to ethanol extraction (3 × 30 L) at RT (room temperature). The resultant was condensed under low pressures, yielding an extract (crude). Further refinement involved extraction with ethyl acetate, resulting in an alkaloid-enriched fraction weighing 157 g. The fraction Fr. I (1.2 g) was purified using RP-18 CC with changing solvent gradient of MeOH-H_2_O (40–80%). This process resulted in the generation of four subpartitions, denoted as Fr. II-1 to Fr. II-4. Subpartition Fr. II-1 (900 mg) subsequently underwent SG (silica gel) CC on a Sephadex LH-20 column, using MeOH as the eluent. This process yielded compound **3** (6 mg), which underwent purification by semi-preparative HPLC using CH_3_CN-0.02% TEA/H_2_O (75:25) to obtain 4 (10 mg). Fraction Fr. II-3 (5.4 g) was processed through SGCC and rinsed with a mixture of PE-EtOAc (15:1). This step yielded constituents 6 (15 mg) and 5 (4.8 mg), with the latter being purified through semi-preparative HPLC using CH_3_CN-0.02% TEA/H_2_O (70:30). Fraction Fr. III (30 g) underwent separation using an RP-18 CC with a MeOH-H_2_O gradient (50–80%), resulting in 3 subpartitions: Fr. III-1–3. Subpartition Fr. III-2 (5.2 g) was analyzed by SGCC, using chloroform-EtOAc (25:1) as the eluting solvent. This process resulted in the extraction of 2 (3.3 mg) and 1 (4.2 mg). Subpartition Fr. III-3 (13 g) was similarly processed, with chloroform-EtOAc (15:1) as the eluent, followed by semi-preparative HPLC using CH_3_CN-0.02% TEA/H_2_O (90:10) to obtain 7 (5 mg). Fraction Fr. IV (30 g) was partitioned using an RP-18 CC with a MeOH-H_2_O gradient (50–80%), resulting in 3 subpartitions: Fr. IV-1–4. Subpartition Fr. IV-2 (4.8 g) was analyzed by SGCC and eluted with a mixture of chloroform-EtOAc (20:1), yielding the major compound **8** (12 mg). This major compound was further analyzed by a Sephadex LH-20 column, eluted with MeOH, leading to the isolation of the major compound **10** (4 mg). Lastly, Subpartition Fr. IV-3 (5.5 g) underwent SGCC with PE-EtOAc (25:1) as the eluent, yielding 9 (12 mg).

#### 3.3.1. Mengtzeanines A (**1**)

White, shapeless powders; [α]: +1.64 (c 1.22, CH_3_OH); UV (CH_3_OH) λmax (log ε = 2.03); IR (KBr) ν_max_: 3437, 2934, 2872, 1702, 1657, 1635, 1454, 1233, 1034 cm^–1^; ESI-TOF/MS: *m*/*z* [M + H]^+^ calculated for C_27_H_41_NO_3_: 428.3159, found: 428.3165; ^1^H-NMR and ^13^C-NMR results (Figure 1, Table 2).

#### 3.3.2. Mengtzeanines A (**2**)

White, shapeless powders; [α]: −3.28(c 1.28, CH_3_OH); UV (CH_3_OH) λmax (log ε = 2.48); IR (KBr) ν_max_: 3436, 2950, 2869, 1460, 1379, 1054, cm^–1^; ESI-TOF/MS: *m*/*z* [M + H]^+^ calculated for C_27_H_43_NO_3_: 430.3316, found: 430.3317; ^1^H-NMR and ^13^C-NMR results (Table 1, Figure 2). 

### 3.4. Cell Culture

Cell line was cultivated in DMEM fortified with 10% FBS and penicillin-streptomycin, and maintained at 37 °C and 5% CO_2_.

### 3.5. MTT Assays

MTT assays were assessed using a prior method with slight modifications [39].

### 3.6. NO Inhibition Assays

NO inhibition assays were assessed using a prior method with slight modifications [40].

### 3.7. ELISA

RAW264.7 macrophages (1 × 10^6^ cells/well) were raised in a platter, followed by overnight incubation at 37 °C. After pre-treatment with Verazine for 2 h, the cells were triggered with LPS (1 μg/mL) for an additional 18 h. Then, 440 μL of RIPA buffer containing 1 mM PMSF was used to treat the lysed cells, and the cells were ruptured immediately on ice. After 30 min, the protein was scraped with a clean tip. The protein was harvested and centrifuged (15,000× *g*, 4 °C, 10 min). The levels of IL1β, IL6, and TNFα were determined using ELISA kits (ABclonal, Technology, Beijing, China).

### 3.8. Western Blot

After pre-treatment with Verazine for 2 h, RAW264.7 macrophages were endured to LPS for an additional 18 h. The cells were ruptured with RIPA buffer comprising 1% phosphatase and protease inhibitors. Protein concentrations were detected using a BCA kit. Subsequently, an equal number of proteins underwent separation through SDS-PAGE and were transferred onto PVDF membranes. Following this, the membranes were subjected to a blocking step at RT with 5% non-fat milk for 60 min. Next, the membranes were kept at 4 °C overnight in the presence of specific primary antibodies. After three washes with TBST, the membranes were exposed to HRP-combined secondary antibodies for 2 h at RT. Detection of immune-blotting signals was accomplished using the BeyoECL Plus electrochemiluminescence reagent [3].

### 3.9. ROS Analysis of Annexin V Expression

RAW264.7 macrophages were primed with Verazine for 2 h, followed by LPS exposure for an additional 18 h. Subsequently, the cells were subjected to two washes with warm PBS and stained for 30 min using a 1 mM DCFH-DA fluorescent probe (Beijing Solarbio Science & Technology, Beijing, China). The stained cells were then examined under an inverted fluorescence microscope (Leica, Wetzlar, Germany).

### 3.10. Statistical Analysis

The data are displayed as mean ± SEM. Statistical tests were conducted via Student’s *t*-test. *p* < 0.05 was deemed significant. All assays were replicated thrice.

## 4. Conclusions

In summary, two novel steroidal alkaloids (**1**, **2**), along with eight known (**3**–**10**) ones, were identified from the roots of *V. mengtzeanum* Loes. The structural properties were determined through extensive NMR spectroscopic analysis. Additionally, Verazine, a steroidal alkaloid, displayed remarkable inhibitory potency against LPS-stimulated NO formation in RAW264.7 macrophages, with an IC_50_ value of 20.41 μM. Moreover, Verazine effectively reduced the release of IL6, TNFα, and IL1β, besides the protein levels of iNOS and COX2 in LPS-activated RAW264.7 macrophages. Furthermore, the reduction in inflammatory mediators is attributed to Verazine’s suppression of NF-κB signal transduction, coupled with its ability to enhance the expression of HO-1 through activation of the Keap/Nrf2 pathway. Consequently, Verazine holds promise as a potential candidate for treating both chronic and acute inflammatory conditions.

## Figures and Tables

**Table 1:** ^1^H and ^13^C NMR data for compound **2** in CDCl_3_.

Position	δC ^a^	δH ^b^	Position	δC ^a^	δH ^b^
1a	37.1	1.79 (1H, m)	14	47.4	1.59 (1H, m)
1b		1.09 (1H, m)	15a	26.6	1.70 (1H, m)
2a	31.8	2.02 (1H, m)	15b		1.36 (1H, m)
2b		1.69 (1H, m)	16	77.9	4.12 (1H, m)
3	71.6	3.52 (1H, m)	17	52.8	2.30 (1H, m)
4a	42.2	2.30 (1H, m)	18	17.4	0.87 (3H, s)
4b		2.23 (1H, m)	19	19.3	1.01 (3H, s)
5	140.6		20	43.1	1.62 (1H, m)
6	121.5	5.34 (1H, d, 6.4)	21	15.7	0.98 (3H, d, 8.4)
7a	32.5	2.03 (1H, m)	22	99.2	
7b		1.33 (1H, m)	23a	28.5	1.54 (1H, m)
8	31.4	1.63 (1H, m)	23b		1.36 (1H, m)
9	43.9	1.30 (1H, m)	24a	28.7	1.78 (1H, m)
10	36.3		24b		1.54 (1H, m)
11a	31.5	1.83 (1H, m)	25	31.0	1.60 (1H, m)
11b		1.49 (1H, m)	26a	50.2	2.72 (1H, m)
12	72.3	3.75 (1H, m)	26b		1.57 (1H, m)
13	44.7		27	19.4	0.85 (3H, d, 7.8)

^a^: 125 MHz, δ in ppm, J in Hz; ^b^: 500 MHz, δ in ppm, J in Hz.

**Table 2:** ^1^H and ^13^C NMR data for compound **1** in CDCl_3_.

Position	δC ^a^	δH ^b^	Position	δC ^a^	δH ^b^
1a	34.0	2.38 (1H, m)	15a	23.9	1.62 (1H, m)
1b		1.59 (1H, m)	15b		1.46 (1H, m)
2a	32.8	2.56 (1H, m)	16a	27.1	1.75 (1H, m)
2b		2.34 (1H, m)	16b		1.58 (1H, m)
3	214.1		17	55.1	1.55 (1H, m)
4a	40.3	2.21 (1H, m)	18	13.4	0.92 (3H, s)
4b		1.35 (1H, m)	19	17.3	1.19 (3H, s)
5	171.3		20	80.0	
6	123.8	5.73 (1H, m)	21	24.7	1.43 (3H, brs)
7	199.6		22	56.0	1.05 (1H, m)
8	55.1	1.73 (1H, m)	23a	20.9	1.54 (1H, m)
9	53.8	0.94 (1H, m)	23b		1.15 (1H, m)
10	38.6		24a	31.9	2.27 (1H, d, 12.8 Hz)
11a	22.1	1.56 (1H, m)	24b		1.83 (1H, m)
11b		1.18 (1H, m)	25	35.0	1.57 (1H, m)
12a	35.7	2.03 (1H, m)	26a	67.7	3.47 (1H, d, 5.9 Hz)
12b		1.70 (1H, m)	26b		1.57 (1H, m)
13	43.8		27	16.5	0.93 (3H, d, 3.4 Hz)
14	56.0	1.20 (1H, m)			

^a^: 150 MHz, δ in ppm, J in Hz; ^b^: 600 MHz, δ in ppm, J in Hz.

## Data Availability

All data generated or analyzed during this study are included in this published article.

## Supplementary Materials

The following supporting information can be downloaded at: https://www.mdpi.com/article/10.3390/molecules28207116/s1, Figures S1.1–S2.9: ^1^ H-NMR, ^13^ C-NMR and DEPT, HMQC, HMBC, COSY, ROESY, HR-ESI-MS, IR, and CD spectrum of Mengtzeanines A and Mengtzeanines B.
