# Supplementary material for: Steroidal Alkaloids from the Roots of Veratrum mengtzeanum Loes. with Their Anti-Inflammatory Activities
Source: Molecules. 2023 Oct 16;28(20):7116. doi: 10.3390/molecules28207116 (PMC10609212; doi:10.3390/molecules28207116)

Steroidal Alkaloids from the Roots of *Veratrum mengtzeanum* Loes. with Their Anti-Inflammatory Activities

Wenjuan Yuan [1,2,†], Jinrong Ma [2,3,†], Xinlan Liu [2,3], Chengting Zi [1,2], Yongkai Xi [1,2], Xiaojing Shen [1], Guodong Li [4], Jun Sheng [2,*] and Xuanjun Wang [2,*]

[1] College of Science, Yunnan Agricultural University, Kunming 650201, China; yuanwj0805@126.com (W.Y.); zichengting@126.com (C.Z.); 2021062@ynau.edu.cn (Y.X.); 2013017@ynau.edu.cn (X.S.)
[2] Key Laboratory of Pu'er Tea, Ministry of Education, Yunnan Agricultural University, Kunming 650201, China; 15752252744@163.com (J.M.); liuxinlan15@163.com (X.L.)
[3] College of Food Science and Technology, Yunnan Agricultural University, Kunming 650201, China
[4] College of Chinese Medicine, Yunnan University of Chinese Medicine, Kunming 650500, China; gammar116@163.com
* Correspondence: shengjunpuer@163.com (J.S.); wangxuanjun@gmail.com (X.W.); Tel.: +886-871-65226191 (X.W.)
† These authors contributed equally to this work.

Supporting information

S1.1 ^{1}H NMR spectrum of **Mengtzeanines A (1)** in $CDCl_3$

S1.2 ^{13}C NMR and dept spectrum of **Mengtzeanines A (1)** in $CDCl_3$

S1.3 HSQC spectrum of **Mengtzeanines A (1)** in $CDCl_3$

S1.4 HMBC spectrum of **Mengtzeanines A (1)** in $CDCl_3$

S1.5 ^{1}H-^{1}H COSY spectrum of **Mengtzeanines A (1)** in $CDCl_3$

S1.6 ROSEY spectrum of **Mengtzeanines A (1)** in $CDCl_3$

S1.7 HRESIMS spectrum of **Mengtzeanines A (1)**

S1.8 IR spectrum of **Mengtzeanines A (1)**

S1.9 CD spectra of **Mengtzeanines A (1)**

S2.1 1H NMR spectrum of **Mengtzeanines B** (**2**) in CDCl3

S2.2 ^{13}C NMR and dept spectrum of **Mengtzeanines B** (**2**) in $CDCl_3$

S2.3 HSQC spectrum of **Mengtzeanines B** (**2**) in $CDCl_3$

S2.4 HMBC spectrum of **Mengtzeanines B** (**2**) in $CDCl_3$

S2.5 ^{1}H-^{1}H COSY spectrum of **Mengtzeanines B** (**1**) in $CDCl_3$

S2.6 ROSEY spectrum of **Mengtzeanines B** (**2**) in $CDCl_3$

S2.7 HRESIMS spectrum of **Mengtzeanines B** (**2**)

S2.8 IR spectrum of **Mengtzeanines B** (**2**)

S2.9 CD spectra of **Mengtzeanines B** (**2**)

S1.1 ¹H NMR spectrum of Mengtzeanines A (1) in $CDCl_3$

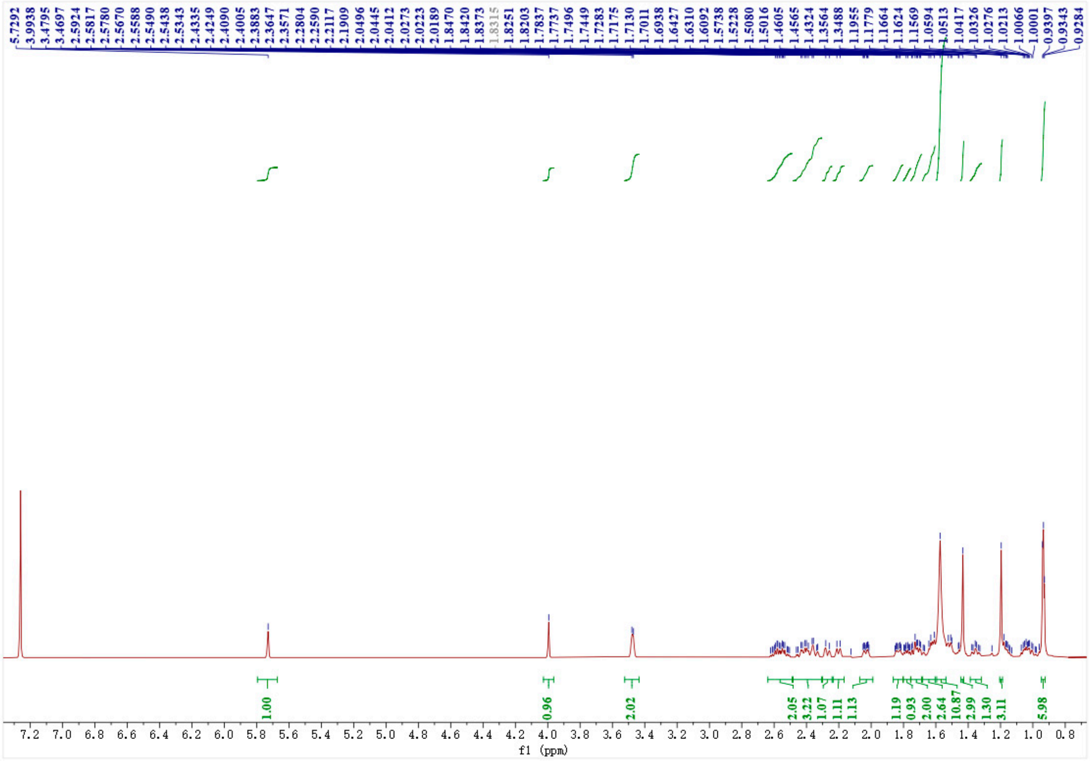

S1.2 ¹³C and dept NMR spectrum of Mengtzeanines A (1) in $CDCl_3$

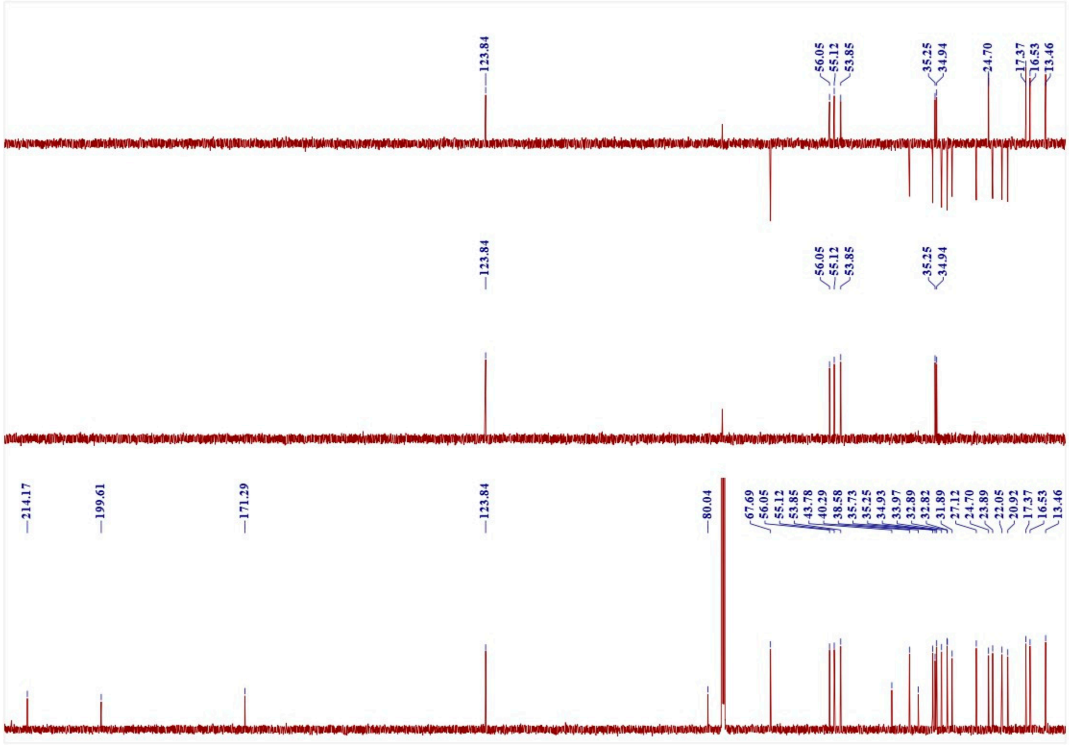

S1.3 HSQC NMR spectrum of Mengtzeanines A (1) in $CDCl_3$

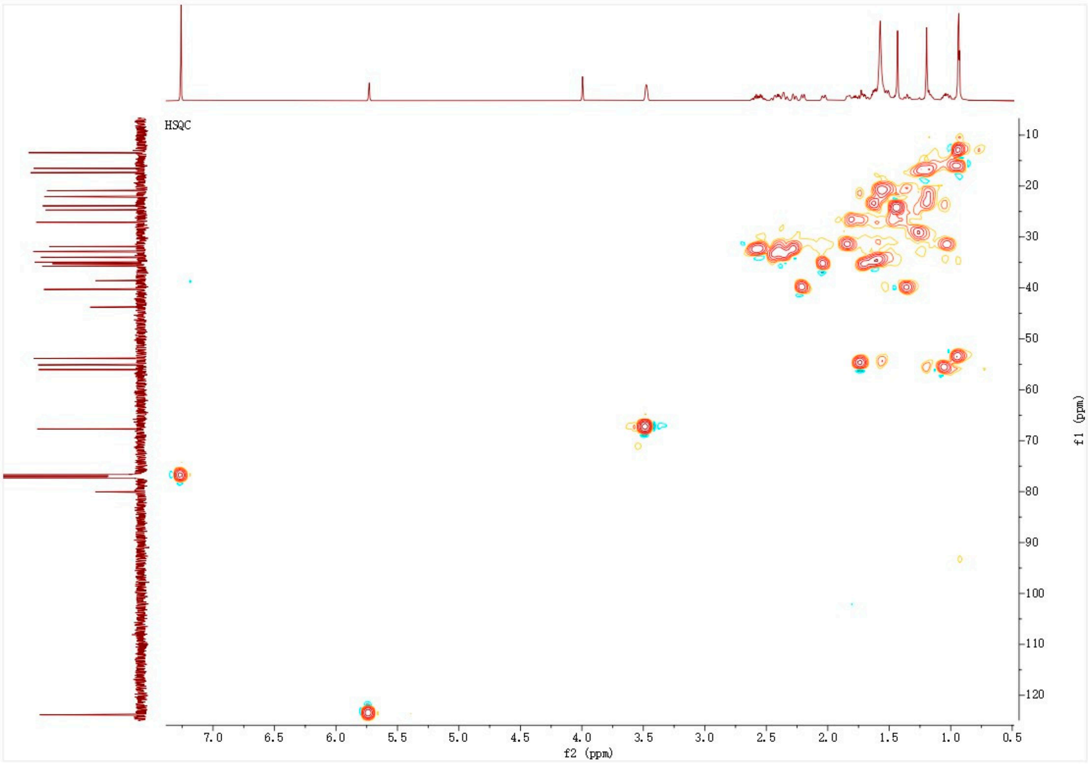

S1.4 HMBC spectrum of Mengtzeanines A (1) in $CDCl_3$

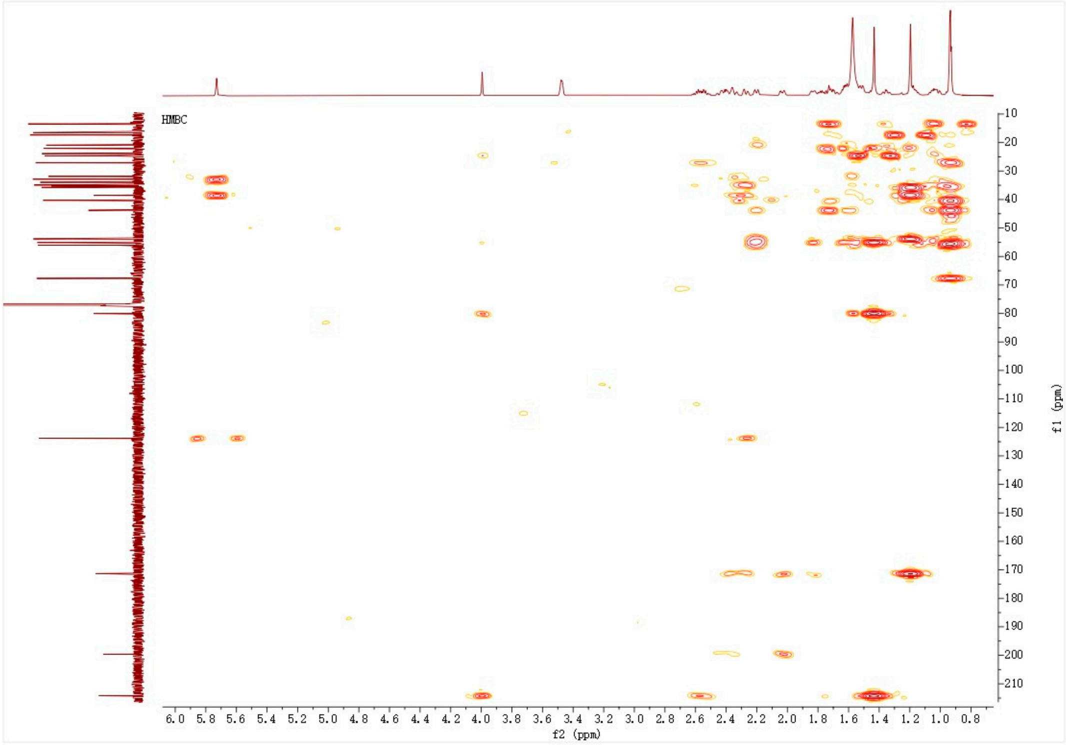

S1.5 ^{1}H-^{1}H COSY NMR spectrum of Mengtzeanines A (1) in $CDCl_3$

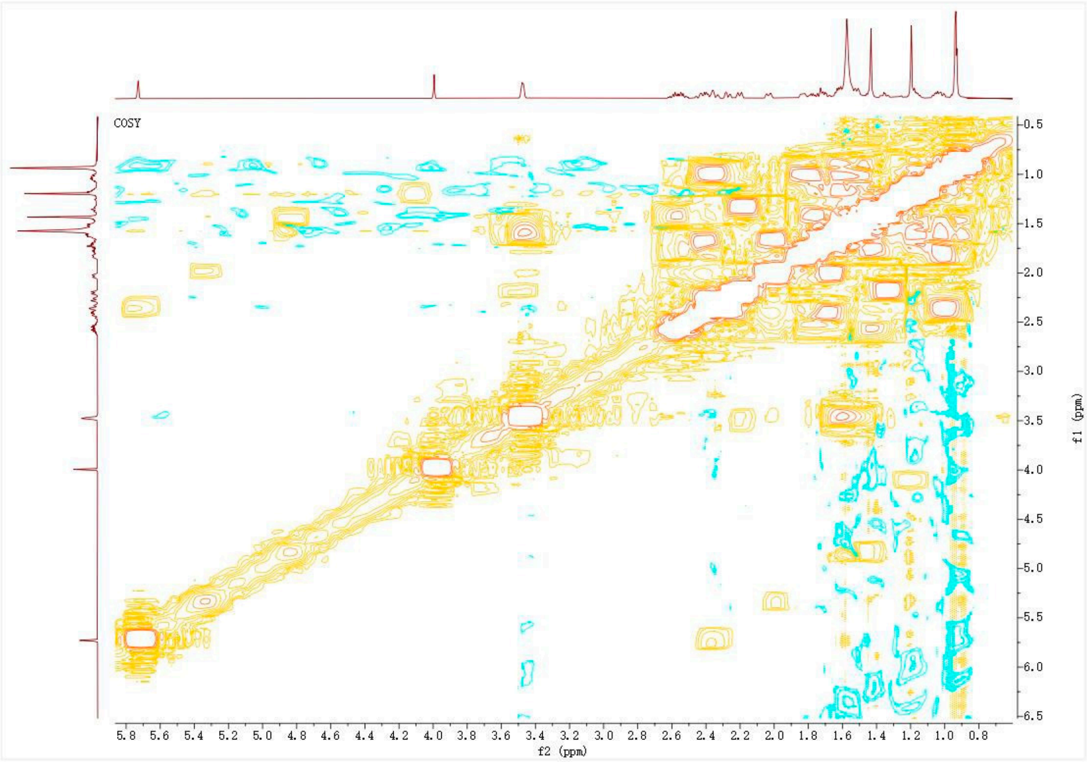

S1.6 ROSEY NMR spectrum of Mengtzeanines A (1) in $CDCl_3$

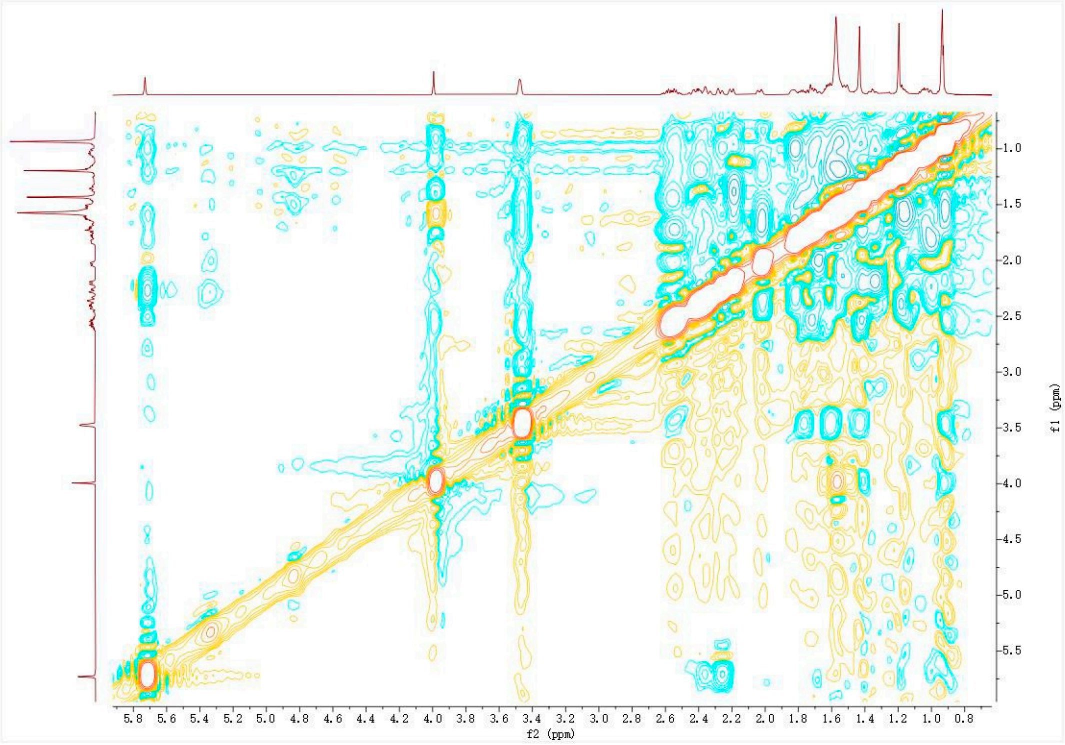

S1.7 HRESIMS spectrum of Mengtzeanines A (1)

Qualitative Analysis Report

**Data Filename**	YJR-43.d	**Sample Name**	YJR-43
**Sample Type**	Sample	**Position**	P1-B1
**Instrument Name**	Instrument 1	**User Name**	
**Acq Method**	s.m	**Acquired Time**	8/19/2022 10:48:05 AM
**IRM Calibration Status**	Success	**DA Method**	PCDL.m
**Comment**			

Sample Group **Info.**
Acquisition SW Version 6200 series TOF/6500 series Q-TOF B.05.01 (B5125.2)

User Spectra

Fragmentor Voltage	Collision Energy	Ionization Mode
135	0	ESI

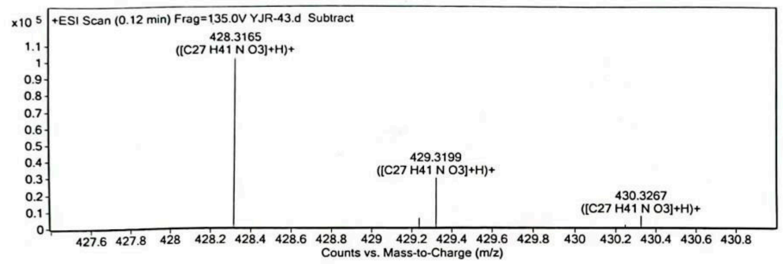

Peak List

m/z	z	Abund	Formula	Ion
174.1593	1	36365.02		
391.2458	1	46051.37		
414.3371	1	49085.23		
426.3003	1	35814.13		
428.3165	1	101767.41	C27 H41 N O3	(M+H)+
429.3199	1	29302.38	C27 H41 N O3	(M+H)+
450.2985	1	51100.77		
453.2987	1	22821.59		
883.6075	1	38146.17		
884.6113	1	19614.52		

Formula Calculator Element Limits

Element	Min	Max
C	3	50
H	0	120
O	0	10
N	0	5

Formula Calculator Results

Formula	CalculatedMass	CalculatedMz	Mz	Diff. (mDa)	Diff. (ppm)	DBE
C27 H41 N O3	427.3086	428.3159	428.3165	-0.60	-1.40	8.0000

--- End Of Report ---

S1.8 IR spectrum of Mengtzeanines A (1)

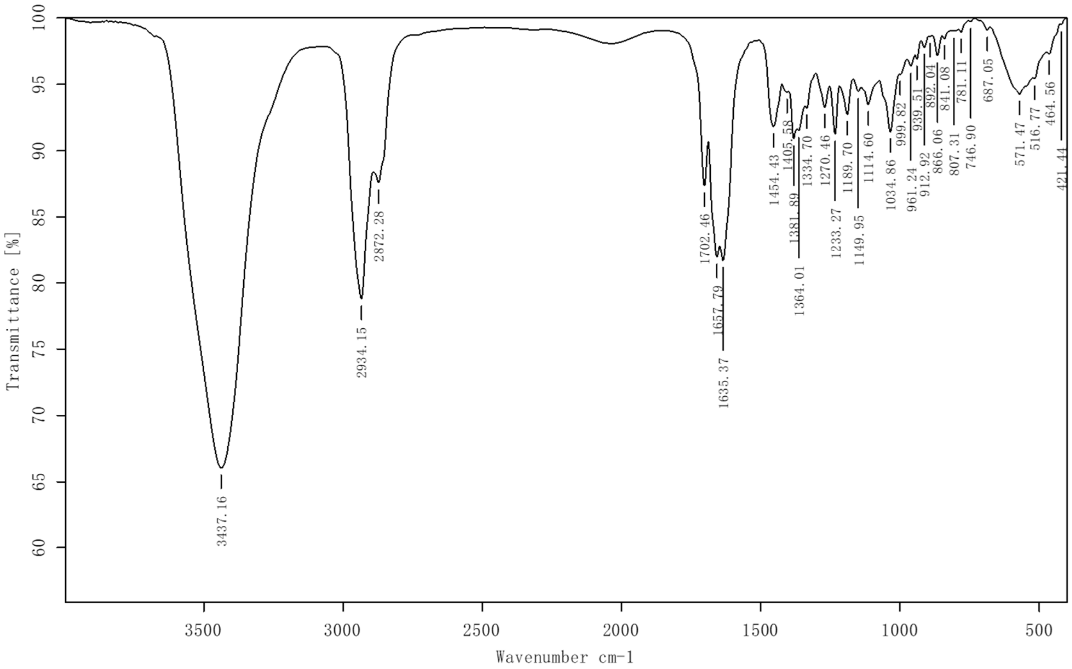

Sample Name: YJR-43	Resolution: 4	Beamsplitter Setting: KBr
Sample Form: KBr	Aperture Setting: 6 mm	Source Setting: MIR
Path of File: E:\data	Number of Background Scans: 16	Instrument Type: BRUKER VERTEX 70
Date of Measurement: 2022/11/24	Number of Sample Scans: 16	Soft Version: OPUS8.1

Figure S1.9 CD spectra of Mengtzeanines A (1)

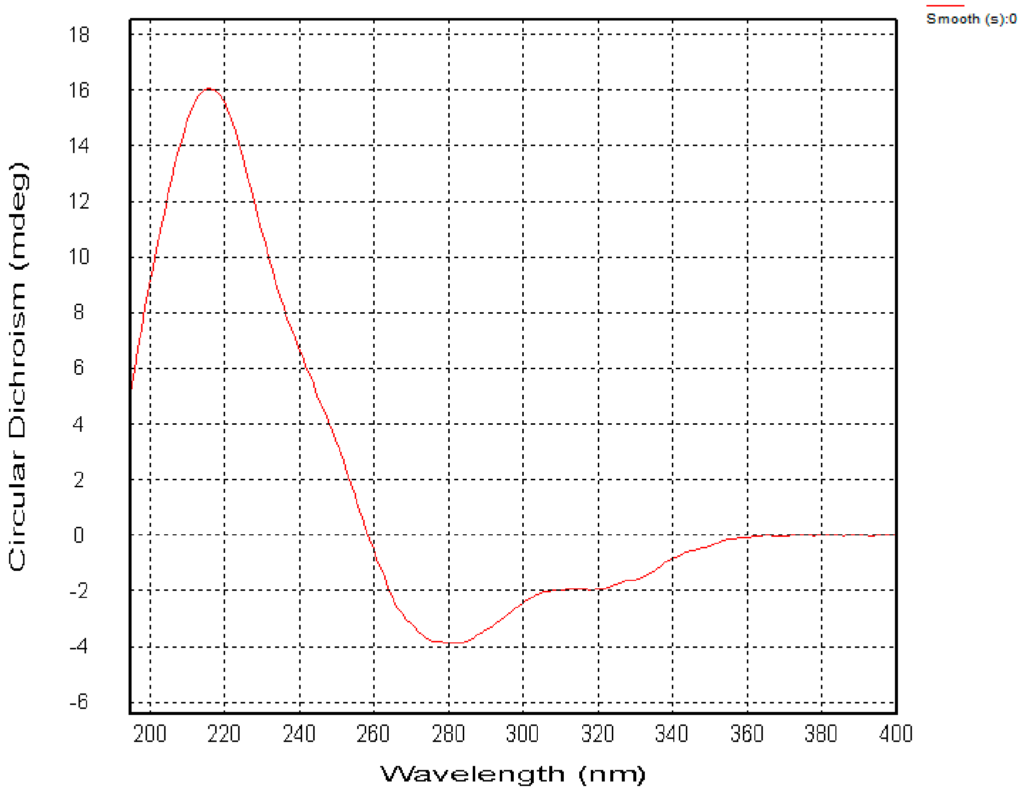

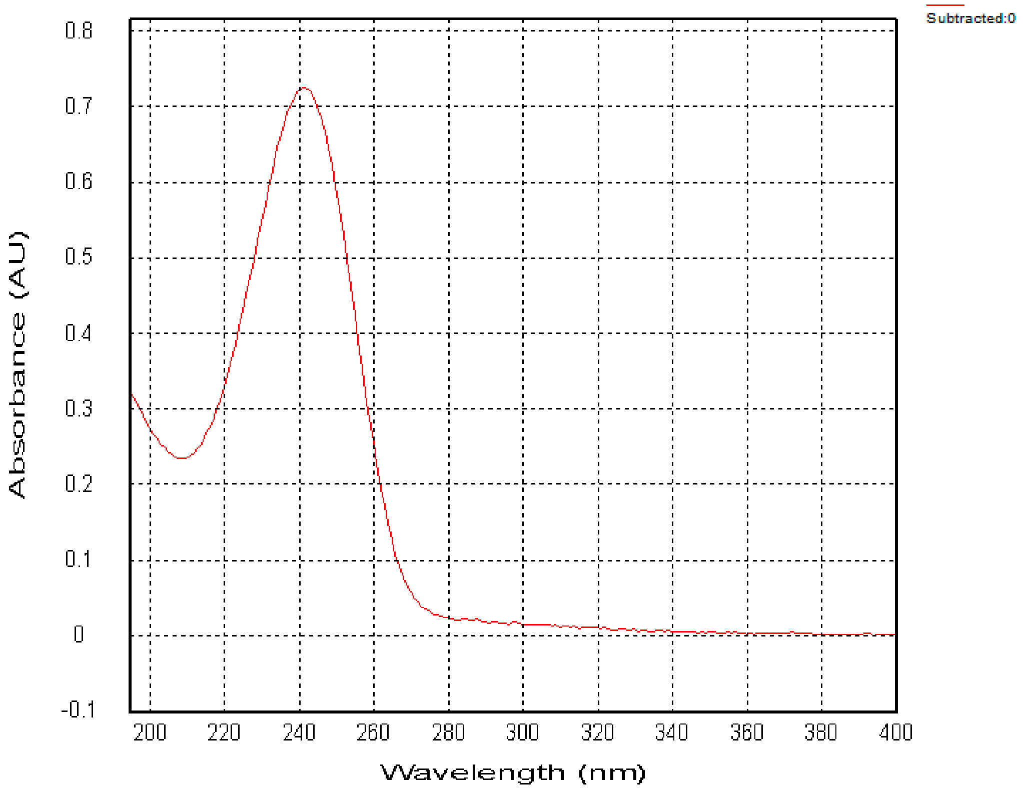

S2.1 ^{1}H NMR spectrum of Mengtzeanines B (2) in $CDCl_3$

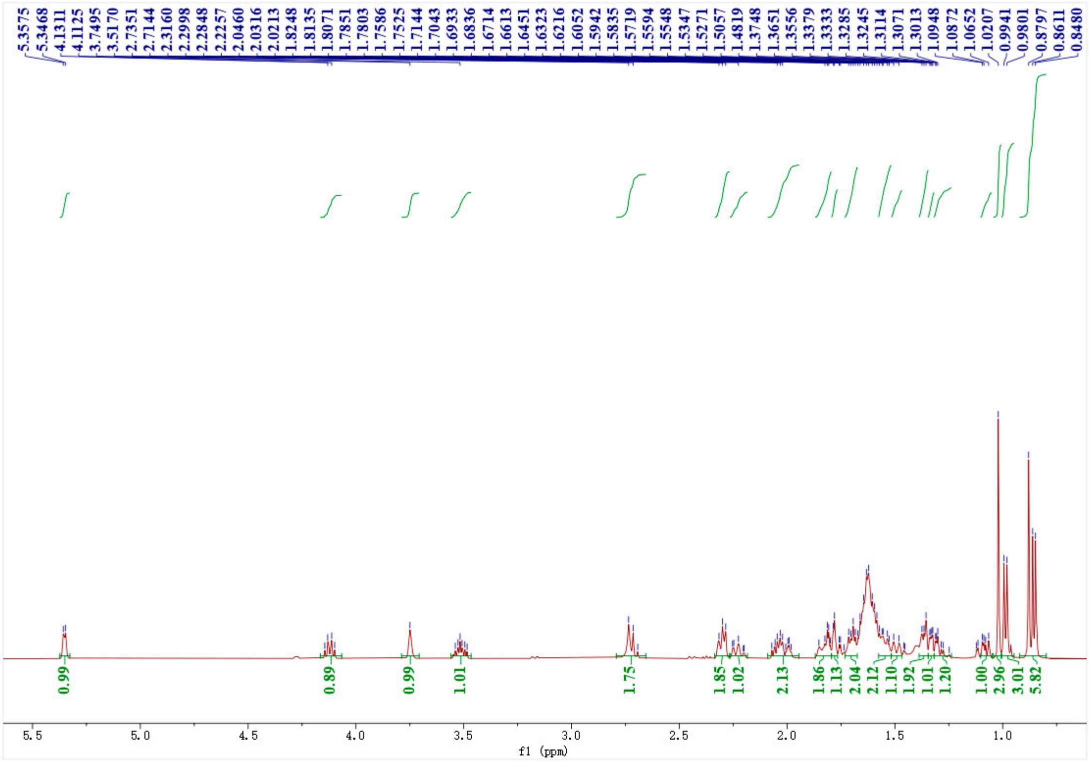

S2.2 ^{13}C and dept NMR spectrum of Mengtzeanines B (2) in $CDCl_3$

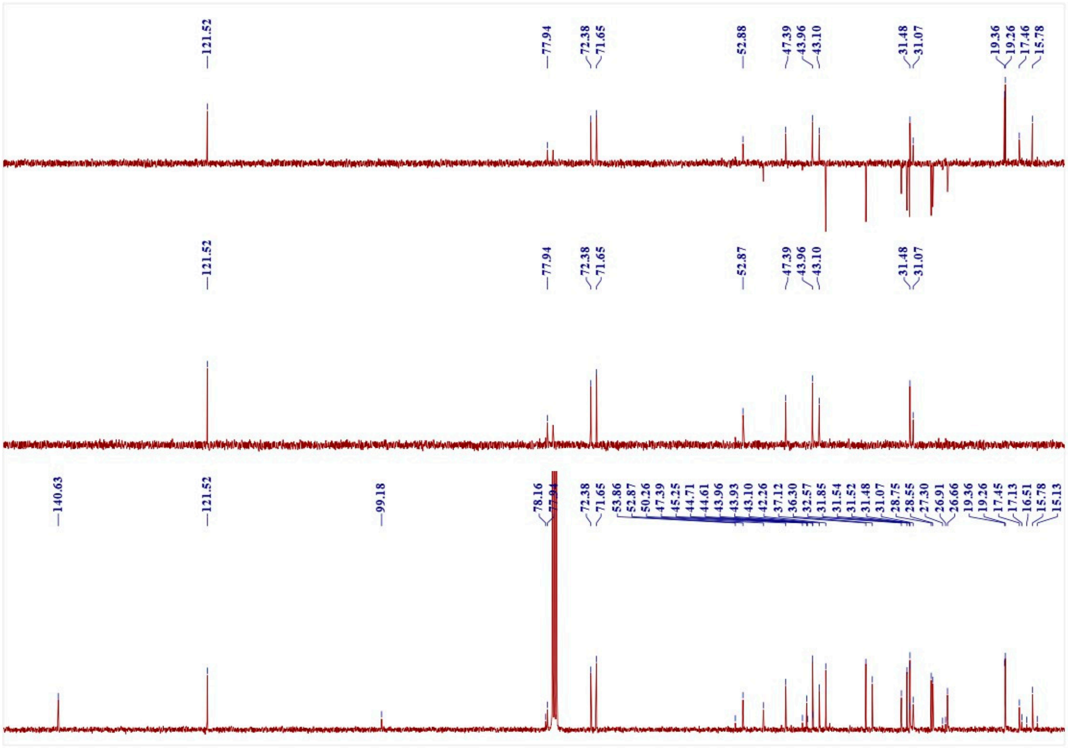

S2.3 HSQC NMR spectrum of Mengtzeanines B (2) in $CDCl_3$

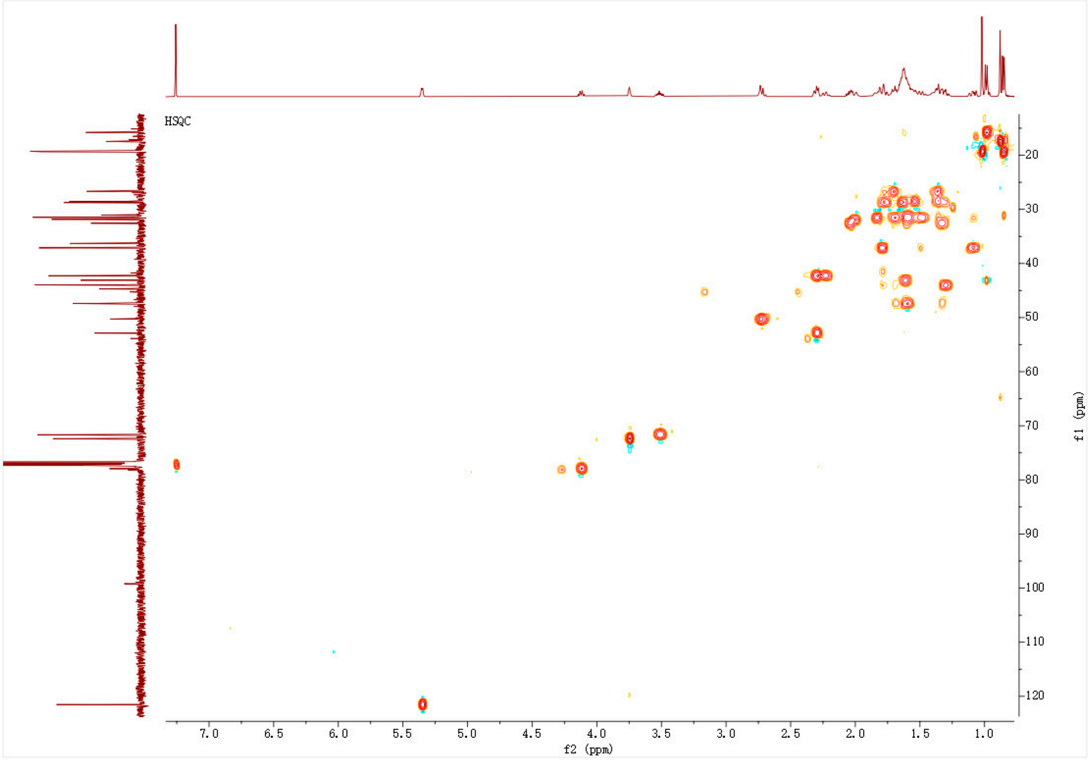

S2.4 HMBC spectrum of Mengtzeanines B (2) in $CDCl_3$

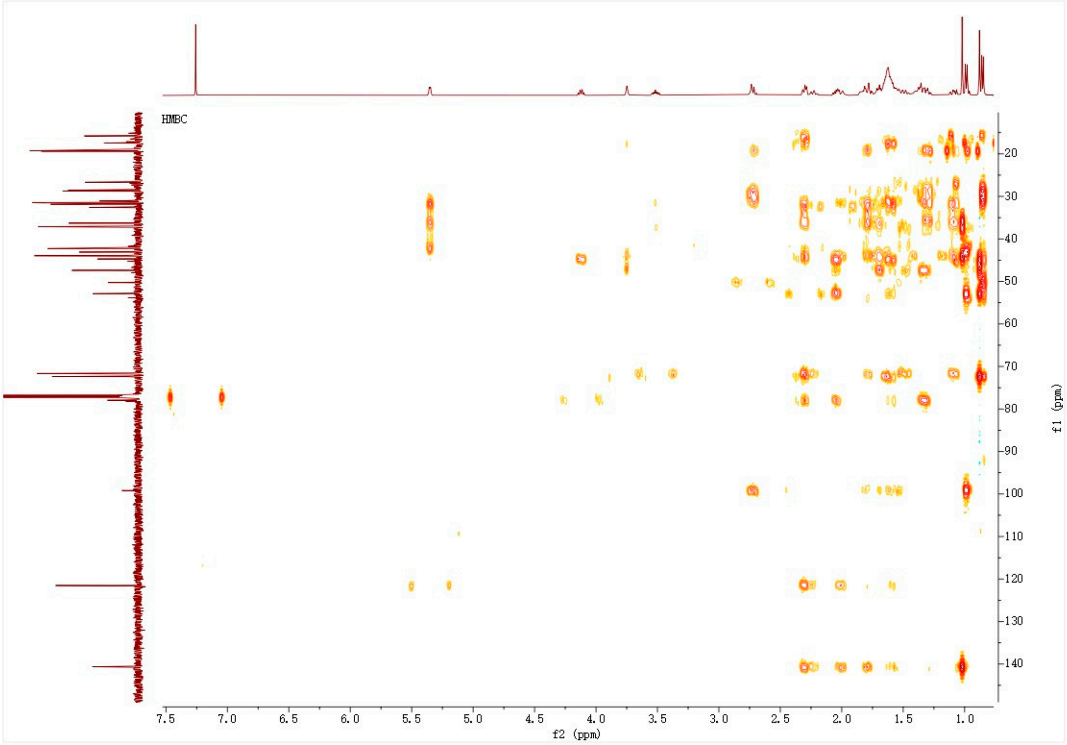

S2.5 ^{1}H-^{1}H COSY NMR spectrum of Mengtzeanines B (2) in $CDCl_3$

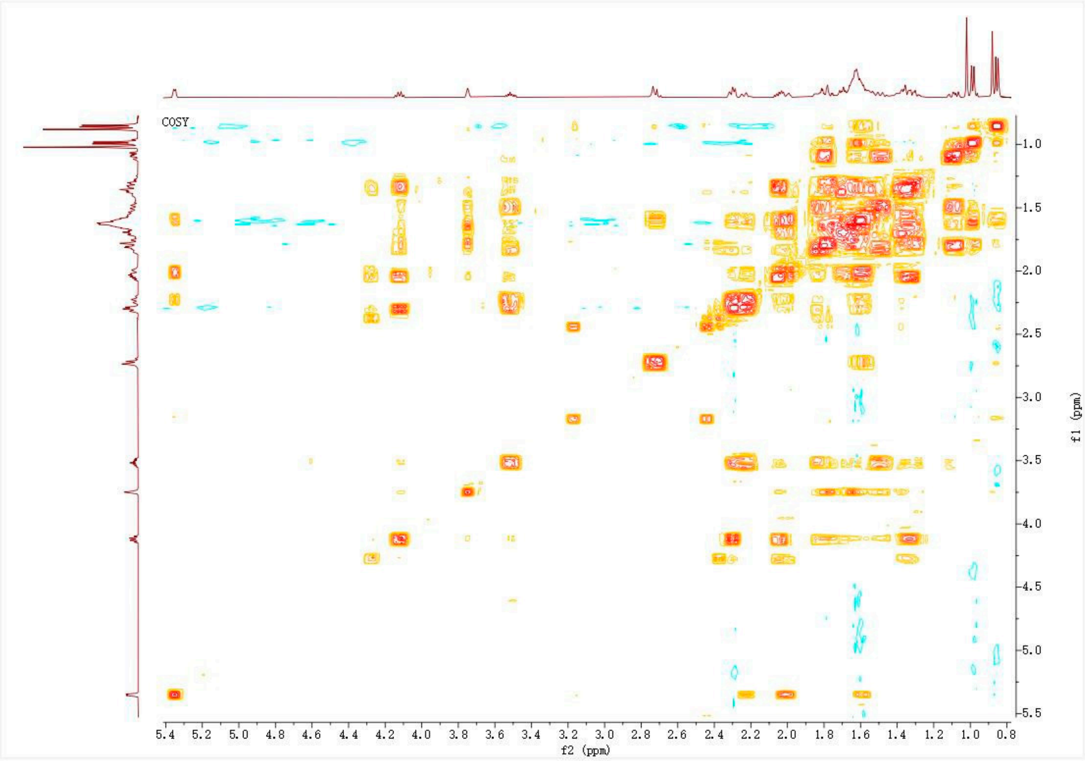

S2.6 ROSEY NMR spectrum of Mengtzeanines B (2) in $CDCl_3$

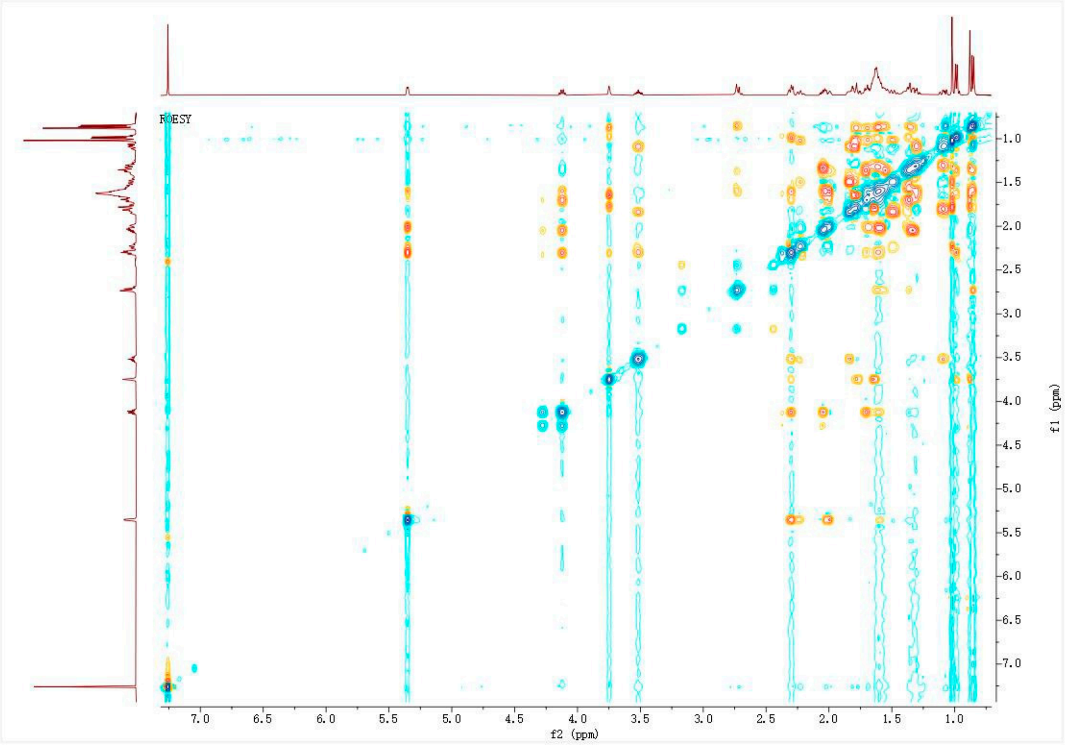

S2.7 HRESIMS spectrum of Mengtzeanines B (2)

Qualitative Analysis Report

**Data Filename**	YJR-27.d	**Sample Name**	YJR-27
**Sample Type**	Sample	**Position**	P1-A6
**Instrument Name**	Instrument 1	**User Name**	
**Acq Method**	s.m	**Acquired Time**	8/2/2022 12:29:38 PM
**IRM Calibration Status**	Success	**DA Method**	PCDL.m
**Comment**			

Sample Group **Info.**
Acquisition SW Version 6200 series TOF/6500 series Q-TOF B.05.01 (B5125.2)

User Spectra

Fragmentor Voltage	Collision Energy	Ionization Mode
175	0	ESI

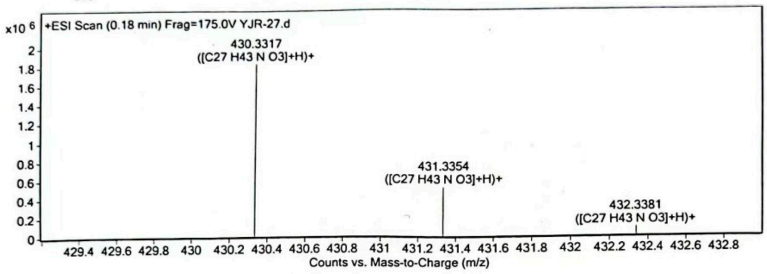

Peak List

m/z	z	Abund	Formula	Ion
114.0911	1	6178.94		
121.0509	1	7999.81		
149.0232	1	7248.5		
223.1231	1	5430.91		
412.3207	1	30274.05		
413.3234	1	8329.93		
430.3317	1	1832789.5	C27 H43 N O3	(M+H)+
431.3354	1	517864.72	C27 H43 N O3	(M+H)+
432.3381	1	82827.16	C27 H43 N O3	(M+H)+
433.3407	1	9408.77	C27 H43 N O3	(M+H)+

Formula Calculator Element Limits

Element	Min	Max
C	3	150
H	0	300
O	0	40
N	0	3

Formula Calculator Results

Formula	CalculatedMass	CalculatedMz	Mz	Diff. (mDa)	Diff. (ppm)	DBE
C27 H43 N O3	429.3243	430.3316	430.3317	-0.10	-0.23	7.0000

--- End Of Report ---

S2.8 IR spectrum of Mengtzeanines B (2)

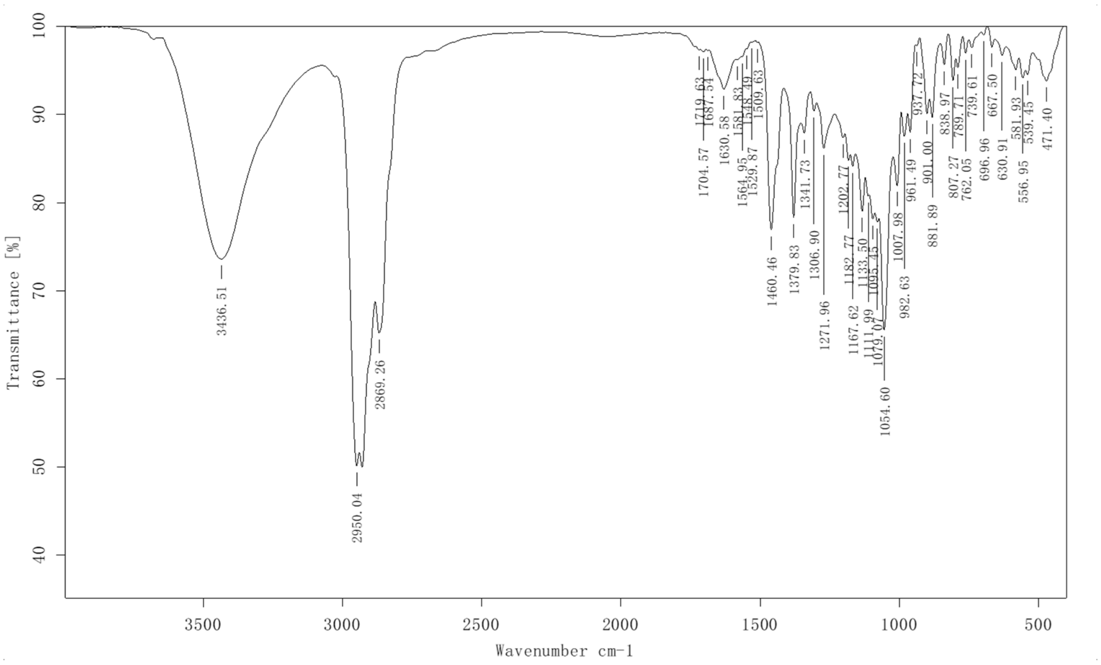

Sample Name: YJR-27	Resolution: 4	Beamsplitter Setting: KBr
Sample Form: KBr	Aperture Setting: 6 mm	Source Setting: MIR
Path of File: E:\data	Number of Background Scans: 16	Instrument Type: BRUKER VERTEX 70
Date of Measurement: 2022/11/24	Number of Sample Scans: 16	Soft Version: OPUS8.1

Figure S2.9 CD spectra of Mengtzeanines B (2)

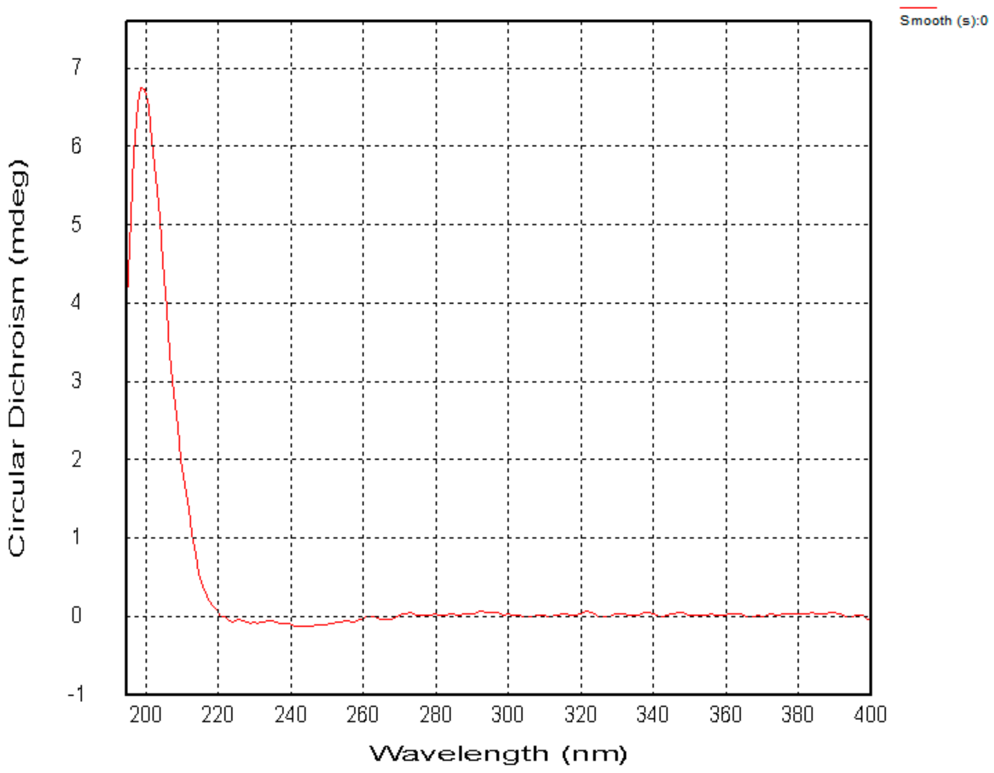

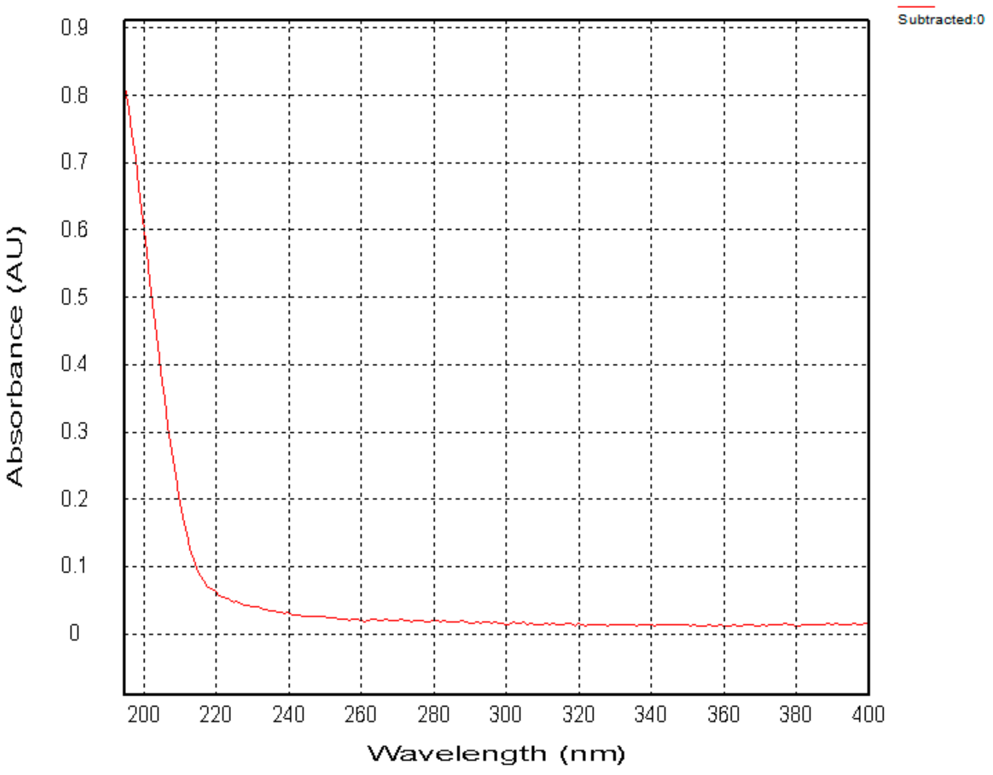

Supplement: Supplementary file 1 [file molecules-28-07116-s001.zip › molecules-2645954-supplementary.pdf]
